# Assessing effective interventions to improve trial retention: do they contain behaviour change techniques?

**DOI:** 10.1186/s13063-020-4151-4

**Published:** 2020-02-21

**Authors:** Eilidh M. Duncan, Tina Bennett, Katie Gillies

**Affiliations:** Health Services Research Unit, Institute of Applied Health Sciences, University of Aberdeen, Aberdeen, UK

**Keywords:** Participant retention, Clinical trials, Interventions, Behaviour change techniques, Theory

## Abstract

**Background:**

Clinical trials often struggle to retain the number of participants required to make valid and reliable assessments about the effectiveness of treatments. Several individual randomised comparisons of interventions to improve retention in trials have been shown to be effective. Many of these retention interventions target participants’ behaviour (e.g. returning questionnaires or attending a follow-up visit). Although not designed as such, these interventions can be thought of as behaviour change interventions. By coding the constituent behaviour change components of effective retention interventions, the interventions’ potential ‘active ingredients’ responsible for improvements in retention can be identified and maximised for future gains.

**Methods:**

Studies reporting effective retention interventions were identified from existing meta-analyses in the literature. Published manuscripts and intervention and comparator group material were coded into their behaviour change techniques (BCTs) using the BCT taxonomy version 1. Two authors independently coded materials using a standardised coding manual and discussed any disagreements to reach consensus. Data on study characteristics including host trial context, timing, mode of delivery and dosage of retention intervention were recorded.

**Results:**

Two intervention types were identified as having evidence of improving retention in existing meta-analyses: monetary incentives and electronic prompts. None of the interventions identified from the included studies explicitly stated a theoretical rationale for their development. BCTs were identified in both intervention and comparator groups across both intervention types and there was heterogeneity with regard to their presentation within and across interventions. The BCTs identified in the ‘monetary incentive’ interventions differed to the comparator group. Contrastingly, the BCTs identified in ‘electronic prompts’ interventions were identical in both the control and intervention groups (within studies) and differed only in terms of mode of delivery and dosing.

**Conclusions:**

Attending a measurement visit or returning a questionnaire is a behaviour and trialists should be mindful of this when designing retention interventions. Our work in this area provides some of the first evidence of the impact of implicit use of BCTs in retention interventions and highlights their potential promise for future trials.

## Background

Clinical trials often struggle to retain the number of participants required on which to make valid and reliable assessments about effectiveness of treatments. It is common for many trial participants (sometimes more than 20%) to drop out before the trial finishes [1]. Moreover, 50% of trials have loss to follow-up of over 11% [1]. The threat of poor retention is a problem for trials and this is further recognised amongst the trials community by its position in joint second as a research priority area for trials methodology [2].

Many strategies are used in an informal way to maximise the numbers of participants staying in a trial to the end, in other words providing outcome data. Yet few strategies to improve retention have robust evidence of effectiveness. Several individual randomised comparisons of interventions to improve retention in trials suggest promise of being effective. However, a Cochrane review of interventions to improve retention to randomised controlled trials revealed that, when pooled in meta-analyses, only a handful of these have a cumulative effect across studies [3]. The 38 trials included within the meta-analysis were grouped into six broad categories of interventions; incentives (monetary and non-monetary), communication strategies (e.g. enhanced cover letter, additional reminders, recorded delivery), questionnaire format (e.g. varying the length or ordering of questions or relevance), participant case management (i.e. increasing support to participants to facilitate retention), behavioural (increasing participants’ motivation) and methodological interventions (i.e. varying aspects of study design such as unblinding treatment allocation) [3]. Only monetary incentives (conditional and unconditional) showed a positive pooled effect on retention in terms of postal and electronic questionnaire response [3]. The effect of other intervention categories was unclear and the review authors concluded more research was required to ascertain effectiveness. Another meta-analysis of interventions to improve retention in RCTs has also been conducted outside of the Cochrane review which highlights the effectiveness of electronic prompts to improve return of postal questionnaires [4].

It is currently unclear whether existing interventions to improve participant retention are based on best evidence, i.e. it is unknown what guided intervention choice, how the intervention was developed and whether a logic model was used to guide interpretation of effect. Moreover, retention within clinical trials involves behaviours; it involves participants completing and returning questionnaires and/or attending site visits, or not. Whilst there is a wealth of theories and evidence about how best to change health behaviour in patients (e.g. promoting health lifestyles), the extent to which interventions targeting retention make use of this evidence is unknown. Identifying and specifying the behaviourally active components of interventions will allow for better understanding of what works (and why) and allow for better replication of successful interventions across different trials testing different clinical interventions in a range of clinical settings.

The aim of this study was to establish whether interventions shown to improve retention to randomised trials are theoretically framed and to identify whether any behaviour change techniques are used (implicitly or explicitly) within these effective interventions.

## Methods

Published reports of interventions shown to be effective (on meta-analysis) at improving retention were reviewed for any reported theoretical underpinning in their development. Intervention descriptions were coded using the Behaviour Change Technique Taxonomy (BCTTv1), which is a hierarchically structured taxonomy of 93 distinct behaviour change techniques (BCTs; the smallest active ingredients of interventions to change behaviour [5]).

### Inclusion criteria

1. Clinical trials that included a nested randomised trial of an intervention to improve retention (i.e return of outcome data)
2. Trials of interventions targeting retention that show evidence of benefit on meta-analysis

### Exclusion criteria

1. Trials of interventions targeting ‘retention’ that did not focus on data collection, e.g. intervention adherence
2. Trials of interventions that were not within a clinical trial setting
3. Trials that solely measured retention as ‘time taken to respond’ rather than ‘response rate’
4. Trials that tested aspects of trial design (e.g. open design) on retention, i.e. had no behavioural component
5. Retention intervention trials that were shown to be individually significant but not evidence at meta-analysis, i.e. individual evaluations

### Search methods for identification of studies

The existing Cochrane review was the main source for interventions examined in this literature review [3]. Of the 38 included trials, we retained those whose trials showed statistically significant findings on meta-analysis. An additional study, known to the authors, which included a meta-analysis of interventions to improve retention in clinical trials (which were not included in the Cochrane review), was also included [4]. A grey literature search (conducted through a citation analysis of the Cochrane review on interventions to improve retention) was also conducted to ensure no additional reviews of retention interventions were eligible for inclusion.

### Selection of studies

Comparisons from the Cochrane review were examined for statistical significance [3]. If the pooled effect of an intervention on sub-group analysis (e.g. monetary incentives) was significant, all trials within that comparison were selected and retention interventions included for further analysis. The Cochrane review identified monetary incentives to be the only effective intervention to improve retention when individual studies were combined in a meta-analysis. Similarly, Clark et al. included a meta-analysis that identified electronic prompts to be effective and these were included in our analysis [4]. The individual studies from each of these meta-analyses were selected for further analysis of interventions.

### Data collection and analysis

Demographic data were extracted from the included studies about the host trial descriptors (clinical population, intervention, time of follow up) and the category of retention intervention (i.e. monetary or communication). Information about group comparisons, target behaviour (i.e. postal questionnaire response rate, clinic visit), condition of incentive of the monetary interventions, sample size and effect size was also recorded.

Corresponding authors of the studies were emailed to request further information about the embedded trial and the retention interventions being tested. Exact content and wording of the intervention (e.g. copies of text messages, emails, cover letters to introduce the intervention and follow-up letters) and the comparator used in the retention trials were requested for coding using the BCTTv1. If the published articles mentioned any theory to inform the development or choice of intervention, this was recorded in the data extraction forms verbatim. The published BCTTv1 (containing 93 individual BCTs [5]) was used to code verbatim descriptions of interventions. A coding manual which included BCT definitions and examples from the published taxonomy and was edited with additional coding rules (developed and agreed by coders ED and TB) was created and used for reference during coding (available from authors by request). Additional coding rules were generated through discussions within the study team (ED, TB and KG) and applied during coding to ensure consistency between and within coders. All data were double coded by ED (a trained and experienced BCT coder) and TB (a trained BCT coder).

BCTs were recorded for both intervention and comparator groups. Both authors compared answers to highlight and resolve discrepancies. Discrepancies that remained despite further discussion were brought to the rest of the research team for group discussion. For each identified BCT, the mode of delivery (e.g. postal, telephone, email) and the dose (i.e. value of intervention and/or how many times it was delivered) was also recorded.

## Results

### Description of studies

A total of seven published retention trials were deemed eligible for inclusion and selected for analysis (see Table 1 for summary of study characteristics). The included studies were conducted in two countries—UK and USA—and published from 2003 to 2015. Retention time points in the host trials also varied, ranging from 2 weeks to 12 months. Each study included between 125 and 2591 participants. The trials in which the embedded studies were set included a range of clinical contexts, such as migraine, chronic obstructive pulmonary disease, back pain, smoking cessation, neck injury, preterm labour and problem drinking. Four trials used or offered a monetary incentive to improve retention [6–9] and three trials focused on electronic prompts to improve trial retention [4, 10, 11]. One included study included two trials of monetary interventions to improve retention; therefore, eight interventions in total were coded from these seven reports [9].

**Table 1:** Characteristics of included studies

Study ID	Year	Country	Clinical population	Timing of follow up	Retention intervention	Timing of retention intervention	Target behaviour	Sample size	Effect size	Explicit use of theory in development?
*Electronic prompts*										
Ashby [10]	2011	UK	Food sensitivity for prevention of migraine	4 week follow up	Electronic prompt: SMS/email/both	Coincide with receipt of questionnaire	Return of postal questionnaire	148	5.4% (95% CI 4.6, 15.4)	No
Clark [4]	2015	UK	Identifying individuals with chronic obstructive pulmonary disease	Between 2 and 6 months (dependent on site)	Electronic prompt: SMS/email/both	Coincide with receipt of questionnaire—‘2 days after the questionnaire was sent’	Return of postal questionnaire	437	8.8% (95% CI 0.11, 17.7)	No
Man [11]	2011	UK	Yoga for treatment of lower back pain	6 months	Electronic prompt: SMS/email	Coincide with receipt of questionnaire—‘on the day that participants were due to receive their questionnaire’	Return of postal questionnaire	125	3% (95% CI 10, 16)	No
*Monetary incentives*										
Bauer [6]	2003	USA	Community intervention trial for smoking cessation	2 weeks	Monetary incentive (cheque for $10 USD or $2 USD)	At time of data collection, i.e. unconditional	Sample collection kits and return of postal questionnaire	300	OR = 1.66 (95% CI 0.83,3.33)	No
Gates [7]	2009	UK	Managing acute whiplash injuries in emergency departments	4 months and 8 months	Monetary incentive (£5 GBP voucher)	At time of data collection—4 months or 8 months, i.e. unconditional	Return of postal questionnaire	2144	RR 1.10 (95% CI 1.05, 1.16)	No
Kenyon [8]	2005	UK	Use of antibiotics to improve neonatal outcomes in preterm labour or preterm rupture of membranes—long term follow up	7 years post trial contact is initiated by letter. Questionnaire sent at 2 weeks	Monetary reward (£5 GBP voucher)	6 weeks post first questionnaire with reminder, i.e. unconditional	Return of postal questionnaire	722	11.7% (95% CI 4.7, 18.6)	No
Khadjesari [9]	2011	UK	Reducing alcohol levels amongst problem drinkers	Study 1: 3 months Study 2: 12 months	Study 1: Monetary incentive 1. £5 Amazon voucher 2. £ 5 charitable donation 3. £250 prize draw Study 2: Monetary incentive (£10 Amazon voucher)	Study 1: promise of incentive within 1 week of questionnaire, i.e. conditional Study 2: promise of incentive at each email reminder, i.e. conditional	Return of online questionnaire—linked in email	Study 1: 1226 Study 2: 2591	Study 1: 2% (95% CI −3, 7) Study 2: 9% (95% CI 5. 12)	No

Clark et al. [4], Ashby et al. [10] and Man et al. [11] looked at the effects of electronic reminders on improving retention rates in returning follow-up postal questionnaires. These studies investigated the effects of electronic prompting (defined by the authors as the intervention coinciding with receipt of the questionnaire) sent via email and/or text message reminders. Control groups did not receive the electronic prompt.

Bauer et al. [6], Gates et al. [7], Kenyon et al. [8] and Khadjesari et al. [9] looked at the effects of what was termed a monetary incentive/reward on improving retention rates to questionnaire return (and return of sample collection kits [6]). Gates et al.[7] and Kenyon et al. [8] randomised participants to receive either a £5 gift voucher or no gift voucher with their postal questionnaire. Bauer et al. randomised participants to either US$10 or US$2, compared to no incentive to improve return of sample collection kits [6]. All three of these trials provided the monetary incentive before the completed questionnaire was received, i.e. receipt of the incentive was not conditional on the behaviour being performed [6–8]. As an online study, Khadjesari et al. included two separate trials within their study[9]. In trial 1 non-responders were randomised to three different interventions—an offer of a £5 Amazon voucher, offer of a £5 donation to Cancer Research UK or offer of a £250 prize draw—or no offer of incentive. After a three-month follow-up, remaining non-responders were further randomised in trial 2 to receive either an offer of a £10 Amazon voucher or no offer of incentive. The offer of the incentive in these evaluations was contingent on the behaviour being performed and was only received when the questionnaire was returned.

None of the embedded trials explicitly mentioned an underlying theory in their development of the interventions to improve retention.

### Behaviour change techniques coding

A summary of BCT coding across all trials is shown in Table 2 and examples of BCT coded content for interventions in Additional file 2. The BCT ‘Social support practical’ (*“advise on, arrange or provide practical help for performance of the behaviour”)* was coded across three trials, for example where a telephone number was provided to participants with the offer of help to complete questionnaires in event of difficulties. ‘Instruction on how to perform a behaviour’ (*“advise or agree on how to perform the behaviour”*) was coded within five trials, for example when participants were informed how to submit their questionnaire responses online. ‘Information about health consequences’ (*“provide information (e.g. written, verbal, visual) about health consequences of performing the behaviour”*) and the related BCT ‘Information about social and environmental consequences (*“provide information about social and environmental consequences of the behaviour”*) was coded across four trials when retention was linked to the overall health/social or environmental question the host trial was trying to answer. ‘Credible source’ (*“present verbal or visual communication from a credible source in favour of or against the behaviour”*) was used within two trials and was coded when letters/reminder letters/emails included an institutional letterhead or were signed by the study coordinator. ‘Adding objects to the environment’ (*“add objects to the environment in order to facilitate performance of the behaviour”*) was coded for five trials, for example when pre-paid envelopes were provided with the questionnaire to facilitate return. The number of BCTs identified within intervention (or comparators) varied across studies, ranging from a minimum of one to a maximum of seven (median of 5).

**Table 2:** Summarised behaviour change technique coding for the interventions tested in the included studies

BCT grouping	BCT reference	BCT name	Trials								Frequency of BCTs across intervention
			*Electronic prompts*			*Monetary incentives*					
			Ashby [10]	Clark [4]	Man [11]	Bauer [6]	Gates [7]	Kenyon [8]	Khad. 1 [9]	Khad. 2 [9]	
Social support	3.2	Social support (practical)		++		++	++				3
Shaping knowledge	4.1	Instruction on how to perform the behaviour		++	++	++	++		++	++	6
Natural consequences	5.1	Information about health consequences		++							1
	5.3	Information about social and environmental consequences					++		++	++	3
Associations	7.1	Prompts/cues	++	++	++	++	++	++	++	++	8
Comparison of outcomes	9.1	Credible source		++			++				2
Reward and threat	10.1	Material incentive (behaviour)			++						1
	10.2	Material reward (behaviour)				+	+	+			3
	10.8	Incentive (outcome)							+	+	2
	10.10	Reward (outcome)							+	+	2
Antecedents	12.5	Adding objects to the environment		++	++	++	++	++			5
Total number of BCTs identified per study			1	6	4	5	7	3	5	5	

++ BCT present in intervention and control

+ BCT present in intervention only

The most commonly coded BCT within both the ‘electronic prompts’ trials and the ‘monetary incentive’ trials was ‘Prompts/Cues’ defined as “*introduce or define environmental or social stimulus with the purpose of prompting or cueing the behaviour. The prompt or cue would normally occur at the time or place of performance*.” An example of this strategy is a letter reminding participants about completing a questionnaire. This letter may be received as part of the control comparator and as part of the intervention (i.e. accompanies the monetary incentive) and the content of which could vary accordingly to also become part of the intervention. This prompting BCT was present across all trials; however, this was present within both control and intervention groups, making it difficult to draw conclusions about any influence on overall effects. The mode of delivery of this BCT employed across trials varied and included email [9], letter [8], phone [6, 7] and multiple modes, including SMS text message[4, 10, 11], as shown in Table 3.

**Table 3:** Mode of delivery and dose of the behaviour change technique prompts/cues across all trials

Trials		BCT - 7.1 Prompts/cues	
		Mode of delivery	Dose
*Electronic prompts*			
Ashby [10]	Intervention group	1) SMS	All once (but unclear)
		2) Email	
		3) SMS and email	
		4) Telephone reminder	
	Control group	1) Telephone reminder	Once
Clark [4]	Intervention group	1) SMS	1) Unclear
		2) Email	2) Unclear
		3) SMS and email	3) Unclear
		4) Written letter	4) Twice
	Control group	1) Written letter	1) Twice
Man [11]	Intervention group	1) SMS	1) Unclear
		2) Email	2) Unclear
		3) SMS and email	3) Unclear
		4) Reminder letters: post	4) Unclear
	Control group	4) Reminder letters: post	1) Unclear
*Monetary incentives*			
Bauer [6]	Intervention group	1) Follow-up calls	1) Unclear
	Control group	1) Follow-up calls	1) Unclear
Gates [7]	Intervention group	1) Telephone reminder	1) U to three
	Control group	1) Telephone reminder	1) Up to three
Kenyon [8]	Intervention group	1) Reminder letter	1) Once
	Control group	1) Reminder letter	1) Once
Khadjesari 1 [9]	Intervention group	1) Email	1) Up to three
	Control group	1) Email	1) Up to three
Khadjesari 2 [9]	Intervention group	1) Email	1) Up to three
	Control group	1) Email	1) Up to three

The monetary incentive strategies used within five interventions are shown in Table 4. When these are coded into BCTs it becomes clear that two of the interventions employed the BCTs ‘Incentive (outcome)’ (*“Inform that a reward will be delivered if and only if there has been effort and/or progress in achieving the behavioural outcome”)* and ‘Reward (outcome)’ (*“arrange for the delivery of a reward if and only if there has been effort and/or progress in achieving the behavioural outcome”*) [9]. For these two interventions, the behavioural outcome is completion of an online questionnaire. For the remaining three trials the intervention’s active ingredient could not be coded directly onto the existing BCT taxonomy [6–8]. As such, it was coded to the closest current match within the taxonomy, which was ‘Material reward (behaviour)’ (*“arrange for the delivery of money, vouchers or other valued objects if and only if there has been effort and/or progress in performing the behaviour”*). However, the monetary rewards in these trials were unconditional upon behaviour. Participants were provided with the incentive regardless of whether they then returned the questionnaire. No BCTs within the current taxonomy clearly capture this kind of unconditional reward.

**Table 4:** Behaviour change techniques, mode of delivery and dose for monetary reward interventions

Trials		BCT - 10.2, Material reward (behaviour)		BCT - 10.8, Incentive (outcome)		BCT - 10.10, Reward (outcome)	
		Mode of delivery	Dose	Mode of delivery	Dose	Mode of delivery	Dose
Bauer [6]	Intervention	1) Postal cheque	1) Once *USD10 cheque or* *USD2 cheque*	x	n/a	x	n/a
	Control	x	n/a	x	n/a	x	n/a
Gates [7]	Intervention	1) postal	1) Once *GBP5 voucher*	x	n/a	x	n/a
	Control	x	n/a	x	n/a	x	n/a
Kenyon [8]	Intervention	1) Postal voucher	1) Once *GBP5 voucher*	x	n/a	x	n/a
	Control	x	n/a	x	n/a	x	n/a
Khadjesari 1 [9]	Intervention	x	n/a	1) Via email	1) Once	1) Via email	1) Once *-Offer GBP5 Amazon voucher* *or* *- Offer of GBP5 CRUK donation* *or* *- Offer of entry into GBP250 charitable prize draw*
	Control	x	n/a	x	n/a	x	
Khadjesari 2 [9]	Intervention	x	n/a	1) Via email	1) once	1) Via email	1) Once *GBP10 Amazon voucher*
	Control	x	n/a	x	n/a	x	n/a

*x* not present, *n/a* not applicable

## Discussion

To our knowledge, we are one of the first research teams to consider retention as a behaviour and to apply a standardised taxonomy to specify the active ingredients of interventions. The interventions included in this study that aimed to improve retention did not explicitly state a theoretical rationale underpinning their development or application. Without this, researchers are limited to pragmatic over theoretically informed solutions. With an explicit theoretical rationale, interventions to improve retention could be more effective and better replicated in other contexts if the mechanism of action was better understood. It should be unsurprising that all retention interventions included in our analysis were identified as including the prompts/cues BCT. For some of the included interventions, this prompting was an explicit planned action of the intervention (e.g. electronic prompts), whereas for others it was an implicit aspect, for example the use of a letter to inform participants of a monetary incentive also doubles as a prompt. Applying a standardised taxonomy to specify the active ingredients of retention intervention strategies has revealed heterogeneity in the active ingredients included and in the modes and dose of delivery of these potentially active ingredients.

The three electronic prompt studies varied with regard to which BCTs were identified, even though the three studies originate from the same research team[4, 10, 11]. The coding we have carried out shows that a variety of active ingredients have been used within one group of ‘communication strategies’, electronic prompts, making it difficult to know which aspects of the intervention may be effective and which not. Furthermore, the trials that have tested these types of strategies have included active ingredients (or BCTs) within both the control and intervention groups, further complicating the picture. It is also interesting to note that of these three studies, the one with the biggest improvement in retention is also the intervention which we identified most BCTs in (for this intervention type). This suggests that other factors, such as varying the dose or mode of delivery of these active ingredients, may also play a role in how effective or not these types of strategies may be. Better specification and reporting of retention interventions would allow for accumulation of knowledge about what works in what circumstances. Using an established taxonomy to do so will enable the specification of ‘standard care’, ensuring that evaluated interventions are distinct from standard care control groups [12].

The active component of providing unconditional monetary interventions could not be accurately coded to the existing BCT taxonomy. We agreed as a team to code it to the closest existing BCT in the taxonomy, which was ‘Material reward (behaviour)’ defined as *“arrange for the delivery of money, vouchers or other valued objects if and only if there has been effort and/or progress in performing the behaviour”*. However, in the unconditional monetary interventions identified in this review, no effort or progress in behaviour was required before the monetary reward was delivered. The other alternative BCTs to code were within the category ‘Incentives’; however, these require participants to be informed in advance of the potential for future reward that is again conditional on effort/progress in behaviour. Clearly neither BCT classification is strictly accurate for the unconditional nature of these monetary interventions. An unconditional monetary intervention could be theorised to influence behaviour in several ways. Receiving money along with a request to return questionnaires may create an expectation of future rewards if participants continue to stay within the trial (and therefore work as an incentive). It may work through creating a social expectation of retention behaviour (e.g. *I’ve been given this money to complete the questionnaire and now it is expected of me that I should*) and working through injunctive norms (which influence behaviour based on what people think is ‘right’ to do based on morals or beliefs) [13]. Further research would be needed in order to compare the effectiveness of conditional and unconditional monetary interventions on retention of participants.

The results from our study suggest that BCTs are already explicitly embedded in retention interventions (and sometimes their comparators). There is now emerging evidence of a role for explicitly embedding BCTs to improve aspects of retention, such as return of postal questionnaires. Evidence from one trial evaluating a theoretically informed letter to target return of trial questionnaires showed a 6% improvement in response rates in the intervention group [14]. Indeed this work is being extended to consider how to actively develop theoretically informed retention interventions that are embedded and produced in participants’ accounts of the barriers to data collection [15]. It is interesting to note that of the seven studies we included in our analysis, only one conducted preliminary work with patient partners to identify which interventions may be most appropriate in their setting [9].

This study has a number of strengths and limitations. We applied this approach to studies included within existing systematic reviews that showed effectiveness through meta-analysis. Future studies may wish to examine the active ingredients of interventions that have been shown not to have an impact on retention and thereby build the evidence about what does not work under what circumstances to better inform development of effective interventions. Behavioural analysis, through BCT coding, of interventions included in Cochrane reviews on both recruitment and retention to trials could make important contributions to our understanding of how interventions targeting potential participants (or trial staff) behaviour does or does not have an effect.

## Conclusions

Given the importance on retaining participants for trial success, considering retention through a behavioural lens may be a fruitful area for trialists wishing to build an evidence base on how to intervene successfully to optimise retention. The BCT-taxonomy used in this study currently has 93 BCTs included, which highlights the variety of potential components that could be tested. In addition, identifying the barriers and facilitators to participant retention and mapping to BCTs hypothesised to change these may also have potential. These tools and approaches could help to inform the design of future retention interventions and enhance the validity of research within this area and the replicability of successful interventions.

## Supplementary information

**Additional file 1.** Word file providing the template data extraction form.

**Additional file 2.** Word file providing example BCT content from retention interventions.

**Additional file 3.** Coding manual.

## Data Availability

The datasets used and/or analysed during the current study are available from the corresponding author on reasonable request.
