# Supplementary figures and images for: Assessing effective interventions to improve trial retention: do they contain behaviour change techniques?
Source: Trials. 2020 Feb 21;21:213. doi: 10.1186/s13063-020-4151-4 (PMC7035706; doi:10.1186/s13063-020-4151-4)

**Additional file 1 – Data extraction form (template)**
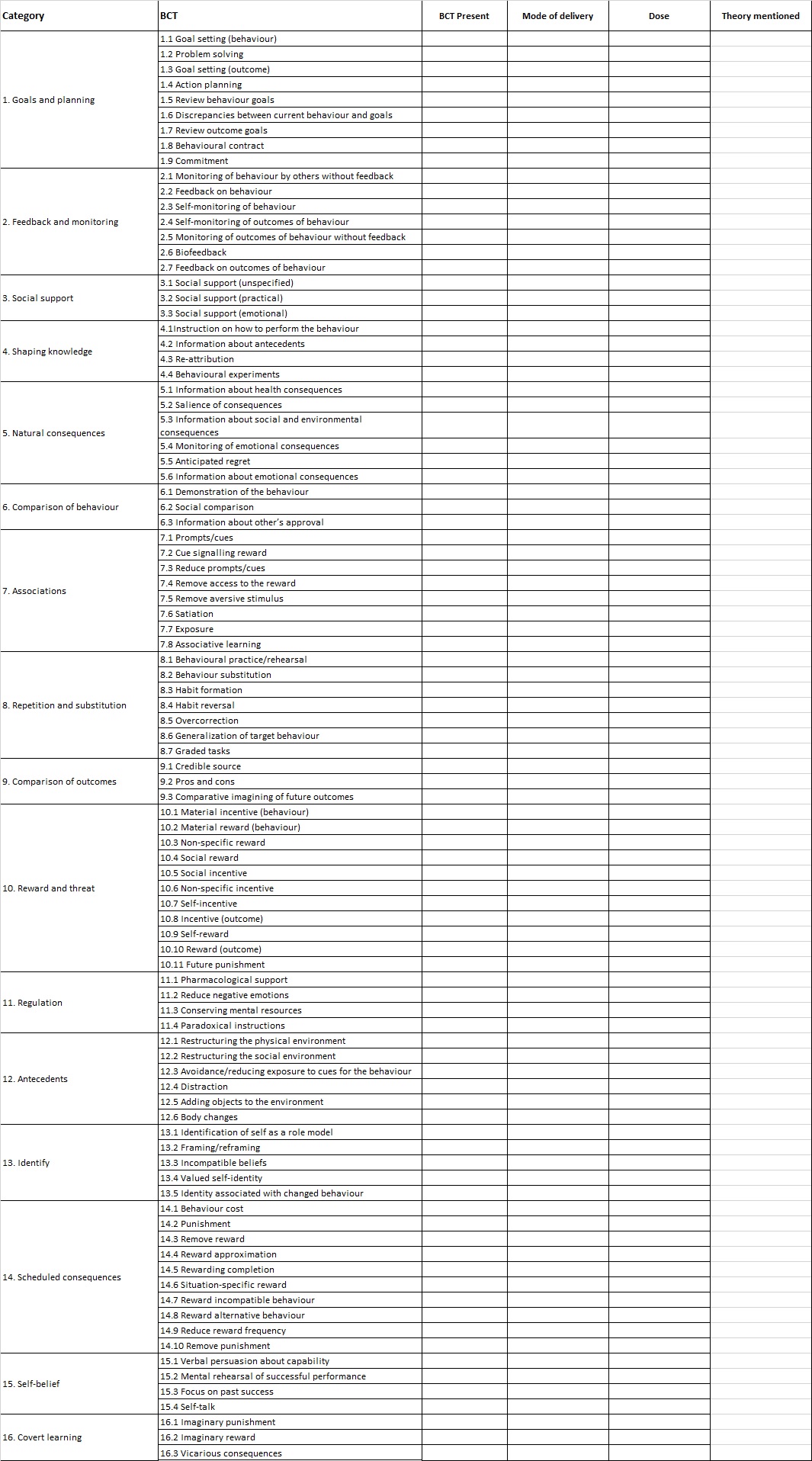

Supplement: Supplementary file 1 — Additional file 1. Word file providing the template data extraction form. [file 13063_2020_4151_MOESM1_ESM.docx]
