# Supplementary material for: Assessing effective interventions to improve trial retention: do they contain behaviour change techniques?
Source: Trials. 2020 Feb 21;21:213. doi: 10.1186/s13063-020-4151-4 (PMC7035706; doi:10.1186/s13063-020-4151-4)
Supplement: Supplementary file 2 — Additional file 2. Word file providing example BCT content from retention interventions. [file 13063_2020_4151_MOESM2_ESM.docx]

**Additional file 2 - Example BCT content from retention interventions.**

| **BCT** | **Content** |
| --- | --- |
| Social support (practical) | ‘If you have any problems completing the questionnaire or if you have any other questions about the study, please contact me [name@uni.co.uk]’ |
| Instruction on how to perform a behaviour | ‘To complete the questionnaire online please follow the URL below and log in using the following details: [webllink]’ |
| Information about health consequences | ‘The study is about the health of your lungs and aims to determine the best approach to identify people who have chronic obstructive pulmonary disease (COPD)’ |
| Information about social and environmental consequences | ‘It is extremely important to the success of this study that you complete these questionnaires, your input is very important as it will help us to understand whether [the trial] is helpful for people like you.’ |
| Prompts/cues | The email/text/letter |
| Credible source | Letterhead with university logo, signature and designation of letter sender |
| Adding objects to the environment | Providing additional copy of questionnaire or freepost envelope |
